# Spontaneous Activity Induced by Gaussian Noise in the Network-Organized FitzHugh-Nagumo Model

**DOI:** 10.1155/2020/6651441

**Published:** 2020-11-24

**Authors:** Qianqian Zheng, Jianwei Shen, Yong Xu

**Affiliations:** ^1^School of Science, Xuchang University, Xuchang, Henan 461000, China; ^2^School of Mathematics and Statistics, Northwestern Polytechnical University, Xi'an, 710072 Shaanxi, China; ^3^School of Mathematics and Statistics, North China University of Water Resources and Electric Power, Zhengzhou 450046, China

## Abstract

In this paper, we show some dynamical and biological mechanisms of the short-term memory (the fixed point attractor) through the toggle switch in the FitzHugh-Nagumo model (FN). Firstly, we obtain the bistable conditions, show the effect of Gaussian noise on the toggle switch, and explain the short-term memory's switch mechanism by mean first passage time (MFPT). Then, we obtain a Fokker-Planck equation and illustrate the meaning of the monostable and bistable state in the short-term memory. Furthermore, we study the toggle switch under the interaction of network and noise. Meanwhile, we show that network structure and noise play a vital role in the toggle switch based on network mean first passage time (NMFPT). And we illustrate that the modest clustering coefficient and noise are necessary to maintain memories. Finally, the numerical simulation shows that the analytical results agree with it.

## 1. Introduction

Short-term memory is a fundamental cognitive function dependent on persistent activity patterns in populations of neurons, which attributes to a fixed point attractor [1]. Persistent activity is represented as a fixed point attractor with multiple stable fixed points [2, 3]. Goldman showed that short-term memory storage was thought to be maintained by persistent neuronal activity when the remembered stimulus is removed [4]. Murray et al. applied the population-level analyses to theoretical neural circuit models to explore potential mechanisms and found that the network connectivity properties play an essential role in uncovering stable population-level working memory representations [5]. Meanwhile, Spaak et al. proposed two mechanisms to underpin the observed dynamic-coding profiles and showed that the primate lateral prefrontal cortex neurons displayed complex dynamics to support stable representations for working memory [6]. Inagaki et al. elucidated persistent activity mechanisms through discrete attractor dynamics and found that perturbations occasionally switched the population dynamics to the other endpoint [7]. Orhan and Ma tried to clarify that sequential and nearly persistent solutions are part of a spectrum [8]. However, some dynamical mechanisms of short-term memory (the fixed point attractor) in neuron activity remain unknown.

For different environments, organisms always try to switch between two states or more, which is vital for the survival in biological systems [9, 10]. Tian and Burrage proposed the toggle switch induced by noise and obtained the role of noise in the genetic toggle switches [11]. Then, Wang et al. illustrated the physiological mechanism in the network-organized systems [12]. And Xu et al. investigated the switch in a genetic toggle system with Lévy noise and showed the influences of the stability index, skewness parameter, and noise intensity on the switch [13, 14]. Wilken and colleagues suggested that neuronal noise was the principal factor that limits the capacity of short-term visual memory [15, 16]. The FitzHugh-Nagumo model (FN) simplifies the Hodgkin-Huxley model, which could describe the dynamical behaviors and phenomena of neurons [17–19]. Meanwhile, noise shows an essential role in the dynamical and biological mechanisms of neurons [20, 21]. Valenti et al. found that the self-correlation of the colored noise causes a reduction of the sufficient noise intensity by analyzing the dynamics of the FN model in colored noise [22]. García-Ojalvo et al. explained a mechanism for sustained signal propagation induced by external fluctuations in bistable media of the FN type [23]. And the coherence resonance of the FitzHugh-Nagumo system under the influence of white Gaussian noise and Lévy noise was investigated [24, 25]. A Fokker-Planck equation for both a single element and a network of globally coupled components was derived in the noisy FitzHugh-Nagumo model [26]. But the toggle switch induced by noise in the network-organized FN model was seldom investigated to explain the dynamical mechanisms of short-term memory (the fixed point attractor).

To illustrate the dynamical and biological mechanism of the fixed point attractor and neurons in short-term memory, we investigate the FN model's bistable state with noise and show how the topology structure and noise play a vital role in the switch of a bistable state. As we all know that short-term memory storage relies on persistent neuronal activity, different kinds of external stimuli always work in the neuronal system, which induces other short-term memory. In this paper, we try to show the effect of Gaussian noise on the toggle switch and try to explain the dynamical mechanism of short-term memory by mean first passage time (MFPT). Then, we illustrate the meaning of the monostable and bistable state in the biological mechanisms. Furthermore, we study the toggle switch under the interaction of network and noise in the network-organized system and show the effect of the external stimulus and topology structure on the short-term memory by NMFPT. Finally, we try to explain the biological mechanism of short-term memory and dynamical mechanism of neurons through the fixed point attractor.

## 2. Model Description

In order to investigate the effect of noise on the switch between rest state (*u*_0_ > 0) and firing state (*u*_0_ > 0), we first consider the dynamical behavior of the following FN model [18]:

(1) $$\begin{array}{c} \begin{array}{c} \frac{\partial u}{\partial t}=g\left(u,\upsilon \right),\\ \frac{\partial \upsilon }{\partial t}=f\left(u,\upsilon \right), \end{array} \end{array}$$

where *u* is the membrane potential, *ν* is a recovery variable, and

(2) $$\begin{array}{c} g\left(u,\upsilon \right)=c\left(eu-\frac{u^{3}}{3}-\upsilon \right),\\ f\left(u,\upsilon \right)=c\left(au-b\upsilon \right). \end{array}$$

The Jacobian matrix at (*u*_0_, *v*_0_) where *f*(*u*_0_, *v*_0_) = *g*(*u*_0_, *v*_0_) = 0 can be expressed as

(3) $$\begin{array}{c} A=\left(\begin{array}{cc} ce-{cu}_{0}^{2} & -c\\ ca & -cb \end{array}\right), \end{array}$$

and the characteristic equation is

(4) $$\begin{array}{c} \left|\lambda E-A\right|=\lambda^{2}+\left(cb-ce+{cu}_{0}^{2}\right)\lambda +cb\left({cu}_{0}^{2}-ce\right)+{ac}^{2}=0. \end{array}$$

Based on the Hurwitz criterion, the system (1) is stable when *cb* − *ce* + *cu*_0_^2^ > 0 and *cb*(*cu*_0_^2^ − *ce*) + *ac*^2^ > 0, namely,

(5) $$\begin{array}{c} u_{0}^{2}>\max\left\{e-b,\frac{be-a}{b}\right\}. \end{array}$$

**Figure 1:**
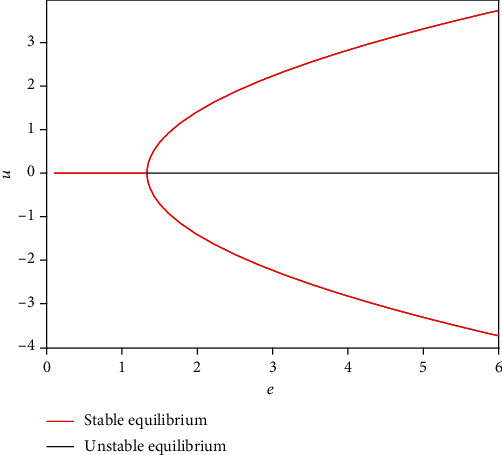
The bifurcation about *e* when *a* = 2, *b* = 1.5, and *c* = 1.

**Figure 2:**
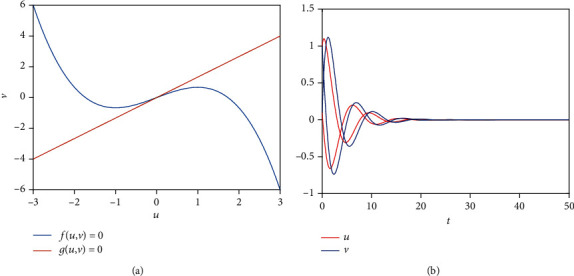
The stability of system (1) when *a* = 2, *b* = 1.5, *c* = 1, and *e* = 1. (a) One equilibrium exists. (b) The system (1) is stable under different initial values.

**Figure 3:**
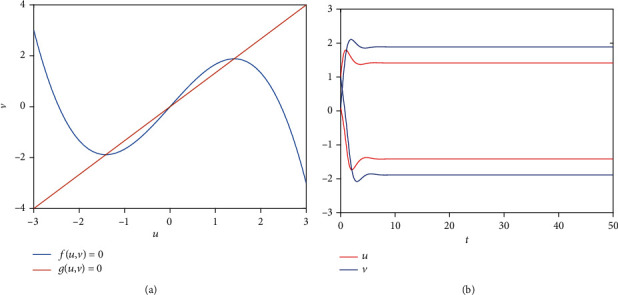
The stability of system (1) when *a* = 2, *b* = 1.5, *c* = 1, and *e* = 2. (a) Three equilibriums exist. (b) The system (1) is bistable under different initial values.

As we all know, *e* plays an important role in the stability of system (1), *u*_0_^2^ = 0 or*u*_0_^2^ = 3(*e* − (*a*/*b*)), and pitchfork bifurcation occurs when *e* = *a*/*b* (Figure 1). And there is an equilibrium point (Figure 2(a)) when 0 < *e* < *a*/*b*, namely, the system (1) is monostable (Figure 2(b)), and the neurons are in a resting state all the time without external stimulus. In general, short-term memory attributes to a fixed point attractor [1]. The short-term memory activities occur and eventually approach steady state when an external stimulus inputs into neurons (Figure 2(b)), which can be treated as the whole process of short-term memory and is consistent with previous results [1, 4]. But resting state and firing state of neurons exist in the neural network. Therefore, we should consider the bistable case in the FN model to investigate some dynamical and biological mechanisms of short-term memory. There are three equilibrium points (Figure 3(a)) when *e* > *a*/*b*, namely, the system (1) is bistable and the equilibrium point (0, 0) can be treated as a critical value of the occurrence of short-term memory (Figure 3(b)). Also, all neurons are in a resting or firing state all the time without external stimulus. Namely, no memory activities occur when the system is in a resting state or firing state (Figure 3(b)), because short-term memory storage was thought to be maintained by persistent neuronal activity when the remembered stimulus is removed [4] and short-term memories are affected by a stable attractor [5, 8]. Here, the bistable system represents two short-term memory points that may exist at the same time when there are different kinds of external input (Figure 3).

## 3. Switch Induced by *α*-Stable Noise

It is well known that a short-term memory occurs when a remembered stimulus inputs into neurons, and short-term memory is maintained by neurons' activity. Here, we treat the remembered stimulus as Gaussian noise and try to uncover Gaussian noise's role in the short-term memory. Firstly, we consider the effect of noise on the switch of the steady state through the following equation:

(6) $$\begin{array}{c} \frac{du}{dt}=f\left(u,\upsilon \right)+\xi \left(t\right),\\ \frac{d\upsilon }{dt}=g\left(u,\upsilon \right), \end{array}$$

where *ξ*(*t*) is the Gaussian noise and the characteristic function is expressed as

(7) $$\begin{array}{c} \phi \left(t\right)=\left\{\begin{array}{c} e^{-\gamma^{\alpha }{\left|t\right|}^{\alpha }\left[1-i\beta \mathrm{sign}\left(t\right)\tan\left(\pi \alpha /2\right)\right]+i\delta t},\;\alpha \neq 1,\\ e^{-\gamma \left|t\right|\left[1-i\beta \mathrm{sign}\left(t\right)\left(2/\pi \right)\log\left|t\right|\right]+i\delta t},\;\alpha =1, \end{array}\right. \end{array}$$

where *α* ∈ (0, 2] (Gaussian noise *α* = 2) is the characteristic exponent. *β* ∈ [−1, 1] is the skewness, the distribution is right-(*β* > 0) or left-(*β* > 0) skewed. Other two parameters are the scale, *γ* > 0, and the location *δ* ∈ *R*.

Events are often triggered when a stochastic process first encounters a threshold, and the time to occurrence of an event is the mean first passage time (MFPT); we adopt the definition from Ref. [27]. As we know that memory occurs when the external stimulus meets a threshold, the weaker stimulation cannot induce any memories. In this paper, a switch occurs from the resting state to the firing state, which means a short-term memory is maintained (loses). A switch occurs from the firing state to the resting state, which also means a short-term memory is lost (or is maintained). To consider the effect of the external stimulus (noise) on MFPT, we consider the backward FPK equation with the region *S* and the boundary*R* and assume

(8) $$\begin{array}{c} P\left(y,t\;|\;x,0\right)=0,\;x\in S,\\ G\left(x,t\right)=\int_{R}dyP\left(y,t\;|\;x,0\right). \end{array}$$

*G*(*x*, *t*) is the probability that the particle remains in *R* at *t* Then,

(9) $$\begin{array}{c} \text{LP}\left(T\geq t\right)=\int_{R}dyP\left(y,t\;|\;x,0\right), \end{array}$$

where LP is the probability that the particle leaves *R*. The backward FPK equation can be described as

(10) $$\begin{array}{c} \partial_{t}P\left(x,t\right)=\Sigma_{i}F_{i}\partial_{i}P+\frac{1}{2}\Sigma_{i,j}g_{ij}\partial_{i}\partial_{j}P, \end{array}$$

where

(11) $$\begin{array}{c} F=\left(\begin{array}{c} f\left(u,\upsilon \right)\\ g\left(u,\upsilon \right) \end{array}\right),\;g=\left(\begin{array}{cc} d & 0\\ 0 & 0 \end{array}\right), \end{array}$$

where *d* = *γ*^*α*^.

For *G*(*x*, *t*), we have

(12) $$\begin{array}{c} \partial_{t}G\left(x,t\right)=\Sigma_{i}F_{i}\partial_{i}G\left(x,t\right)+\frac{1}{2}\Sigma_{i,j}g_{ij}\partial_{i}\partial_{j}G\left(x,t\right), \end{array}$$

where the initial condition is

(13) $$\begin{array}{c} G\left(x,0\right)=\left\{\begin{array}{c} 1,\;x\in R,\\ 0,\;x\in R^{c}, \end{array}\right. \end{array}$$

and the boundary condition is

(14) $$\begin{array}{c} G\left(x,t\right)=0,\;x\in S. \end{array}$$

Finally, MFPT can be defined as

(15) $$\begin{array}{c} T\left(x\right)=\int_{0}^{\infty }G\left(x,t\right)dt. \end{array}$$

According to the formula between the FPK equation and stochastic differential equation [27], we obtain

(16) $$\begin{array}{c} \partial_{t}P=-\Sigma_{i}\partial_{i}\left(F_{i}P\right)+\frac{1}{2}\Sigma_{i,j}\partial_{i}\partial_{j}\left(G_{ij}P\right), \end{array}$$

where

(17) $$\begin{array}{c} F=\left(\begin{array}{c} f\left(u,\upsilon \right)\\ g\left(u,\upsilon \right) \end{array}\right),\\ G\left(\begin{array}{cc} d & 0\\ 0 & 0 \end{array}\right). \end{array}$$

**Figure 4:**
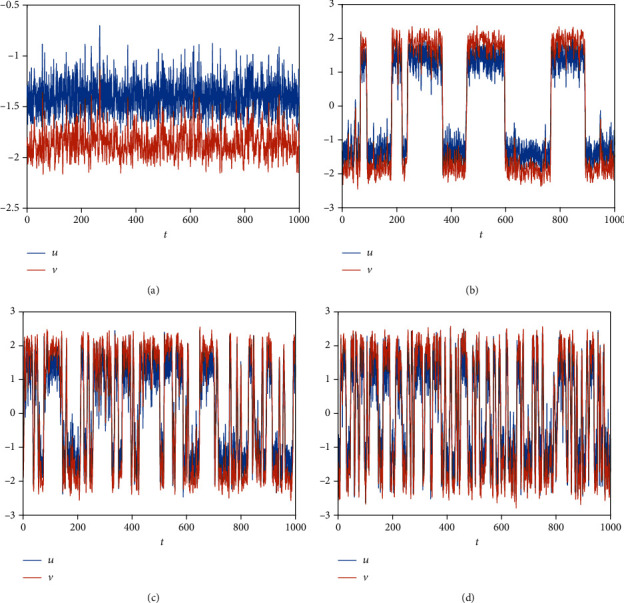
The steady state of system (21) when *a* = 2, *b* = 1.5, *c* = 1, *e* = 2, *α* = 2, *β* = 0, *δ* = 0, and *d* = *γ*^*α*^: (a) the steady state when *γ* = 0.1; (b) the steady state when *γ* = 0.2; (c) the steady state when *γ* = 0.3; (d) the steady state when *γ* = 0.4.

As we all know, neurons' state will vary (like a switch occurs) when a short-term memory occurs. From Figure 4, the steady-state switch does not occur (a short-term memory occurs and is not lost) when the noise strength is small (Figure 4(a)). Namely, a weaker remembered stimulus can induce a short-term memory because of the persistent neuronal activity near a stable attractor [4, 5, 8]. And the switch occurs (a short-term memory is lost and another one memory occurs) (Figure 4(b)) when the noise intensity increases. With the noise strength increasing, the switch frequency becomes faster and faster (Figure 4(c)), and even retention time tends to zero (Figure 4(d)). Namely, the stronger the external stimulus, the shorter the short-term memory, consisting of the actual situation in life. The above phenomenon also means that an appropriate stimulus is necessary to switch neurons to maintain a short-term memory.

**Figure 5:**
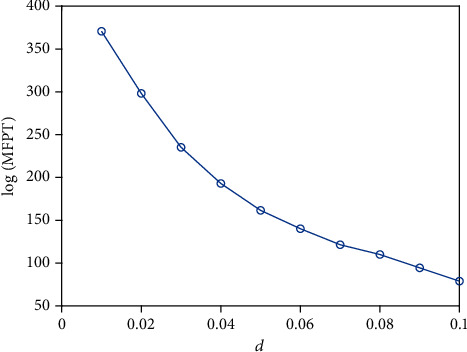
The mean first-passage time about *d* = *γ*^*α*^ when *a* = 2, *b* = 1.5, *c* = 1, *e* = 2, and initial value (*u*_0_, *ν*_0_) = (−1.414, −1.885).

**Figure 6:**
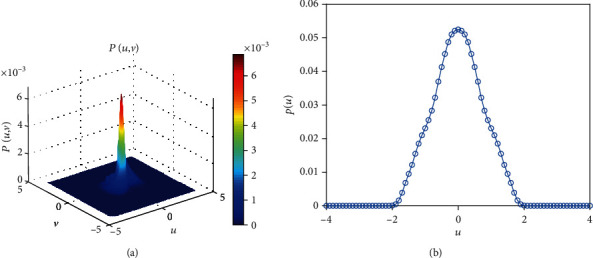
The joint probability density function about *d* when *a* = 2, *b* = 1.5, *c* = 1, *e* = 1, and *d* = 0.04.

**Figure 7:**
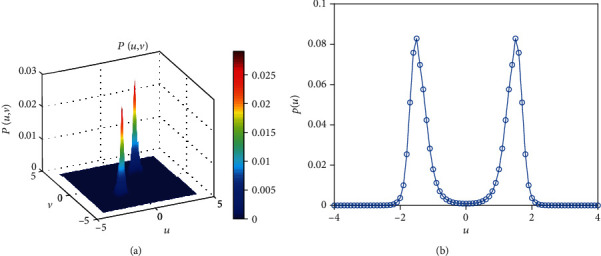
The joint probability density function about *d* when*a* = 2, *b* = 1.5, *c* = 1, *e* = 1, and *d* = 0.04.

MFPT is an important measurement tool to determine whether a short-term memory occurs. The intensity of external stimulus could explain how easy it (a short-term memory occurs) will be. From our simulation (Figure 5), the MFPT decreases with the increase of the noise strength. Namely, the more intense the stimulus, the easier the short-term memory switch, and MFPT is the retention of short-term memory. Finally, the probability density function is given (Figures 6 and 7). As the parameter *e* varies, the monostable state (Figures 6(a) and 6(b)) or bistable state (Figures 7(a) and 7(b)) exists in the system. Meanwhile, a short-term memory relies on the idea of a fixed point attractor [5, 8], namely, the existence of a short-term memory or two short-term memories is possible in the brain, and a stable attractor means a short-term memory point. In general, a short-term memory should be kept for a while, rather than quickly disappear, which means that an appropriate stimulus is necessary to maintain a short-term memory. And a switch for a neuron means a short-term memory loses, and another short-term memory occurs.

## 4. The Network-Organized FN Model with Noise

As we all know, the nervous system consists of neural networks, and a switch occurs (a short-term memory is lost and another one short-term memory occurs) when an external stimulus is involved. A general network-organized Fitzhugh-Nagumo model with noise can be written as

(18) $$\begin{array}{c} \frac{{du}_{i}}{dt}=f\left(u_{i},\upsilon_{i}\right)+d_{1}\Sigma_{j}L_{ij}u_{i}+\xi_{i}\left(t\right), \end{array}$$

*dυ*_*i*_/*dt* = *g*(*u*_*i*_, *υ*_*i*_) + *d*_2_*Σ*_*j*_*L*_*ij*_*υ*_*i*_, where *L* is the Laplacian matrix of the nearest-neighbor coupled network [28], *d*_1_, *d*_2_ are the coupling strength, and *ξ*_*i*_(*t*)(*i* = 1, ⋯, *N*) is the Gaussian noise.

In order to investigate the MFPT in the network-organized FN model, we define the network MFPT (NMFPT) as the following [13]:

(19) $$\begin{array}{c} \tau =\min\left\{\tau \left(\text{high}\right),\tau \left(\text{low}\right)\right\}, \end{array}$$

where

(20) $$\begin{array}{c} \tau \left(\text{high}\right)=\inf\left\{t:u_{i}\left(t\right)>u_{\text{high}}\right\},\\ \tau \left(\text{low}\right)=\inf\left\{t:u_{i}\left(t\right)>u_{\text{high}}\right\}.\\ \text{NMFPT}=E\left[\tau \right]. \end{array}$$

First, we consider the effect of network on the stability of the network-organized FN model, namely,

(21) $$\begin{array}{c} \frac{{du}_{i}}{dt}=f\left(u_{i},\upsilon_{i}\right)+d_{1}\Sigma_{j}L_{ij}u_{i}, \end{array}$$

*dυ*_*i*_/*dt* = *g*(*u*_*i*_, *υ*_*i*_) + *d*_2_*Σ*_*j*_*L*_*ij*_*u*_*i*_.

**Figure 8:**
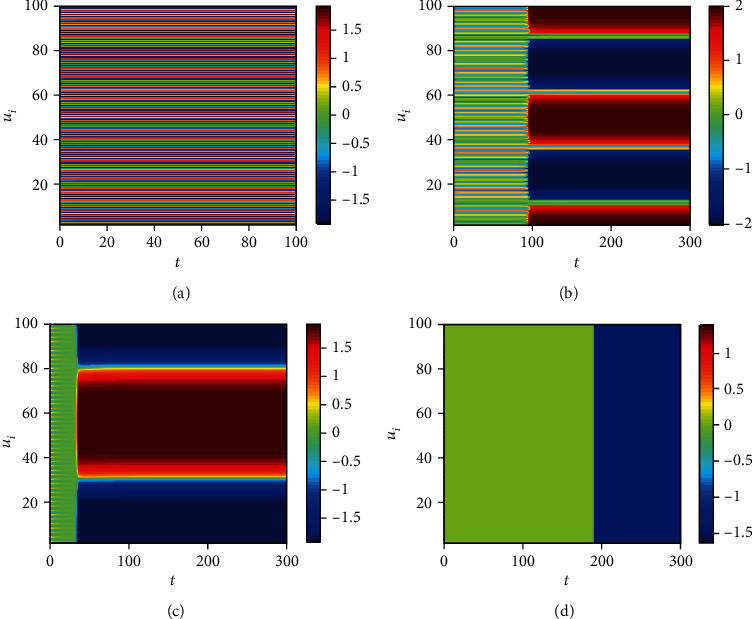
The distribution of firing about the clustering coefficient (cc): (a) *cc* = 0; (b) cc = 0.6923; (c) cc = 0.7105; (d) cc = 0.7286.

**Figure 9:**
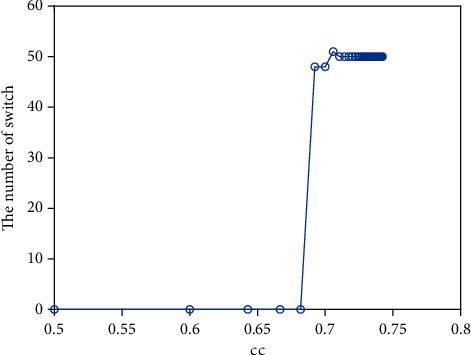
The number of switch about clustering coefficient (cc).

The clustering coefficient is a critical evaluation index of the nearest-neighbor coupled network, which shows the current connectivity and network's main characteristic. Because the system is bistable and the initial value is different, the system will tend towards the other steady state (Figure 8(a)) when the clustering coefficient cc = 0. And the switch occurs (Figure 8(b)) when the clustering coefficient increases, and the occurrence of a switch is different (Figures 8(b)–8(d)) because of the clustering coefficient. Assume a node represents a neuron; the persistent activity of a neuron means memory is maintained. A short-term memory is lost when the neuron is back to the other state. A short-term memory sometimes requires multiple neurons to work together, such as 100 short-term memories in 100 neurons (Figure 8(a)), 5 short-term memories in 100 neurons (Figure 8(b)), 3 short-term memories in 100 neurons (Figure 8(c)), 1 short-term memory in 100 neurons (Figure 8(d)). Namely, some neurons are necessary to maintain short-term memory, rather than a neuron. And the number of neurons to keep a short-term memory is different, which is related to the clustering coefficient. Namely, the number of neurons for everyone to maintain a short-term memory may be different. Meanwhile, the number of switch cases is different according to the clustering coefficient (Figure 9). Namely, memories have something to do with the character of the neuronal network under some initial conditions. Also, the modest clustering coefficient is necessary for the maintaining of memories.

**Figure 10:**
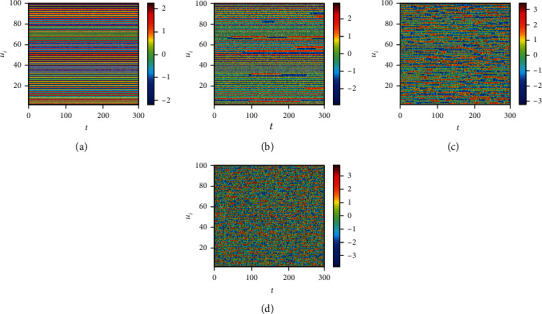
The switch of steady state under different conditions when cc = 0: (a) *γ* = 0.1; (b) *γ* = 0.4; (c) *γ* = 0.6; (d) *γ* = 0.9.

**Figure 11:**
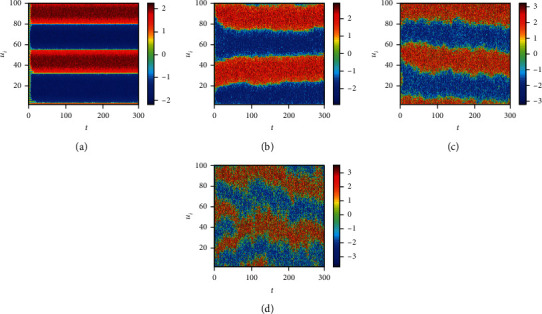
The switch of steady state under different conditions when cc = 0.6923; (a) *γ* = 0.1; (b) *γ* = 0.4; (c) *γ* = 0.6; (d) *γ* = 0.9.

**Figure 12:**
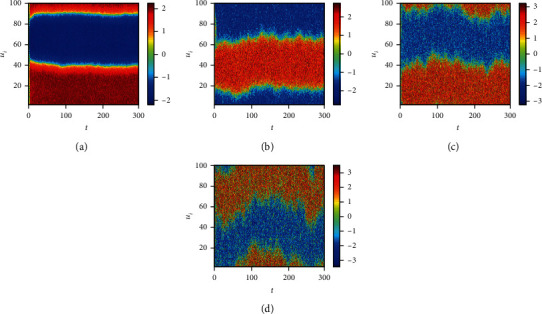
The switch of steady state under different conditions when cc = 0.7105: (a) *γ* = 0.1; (b) *γ* = 0.4; (c) *γ* = 0.6; (d) *γ* = 0.9.

**Figure 13:**
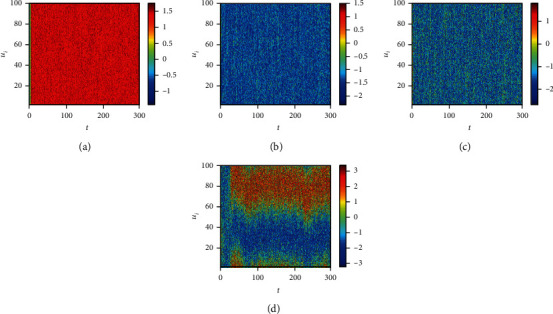
The switch of steady state under different conditions when cc = 0.7286: (a) *γ* = 0.1; (b) *γ* = 0.4; (c) *γ* = 0.6; (d) *γ* = 0.9.

**Figure 14:**
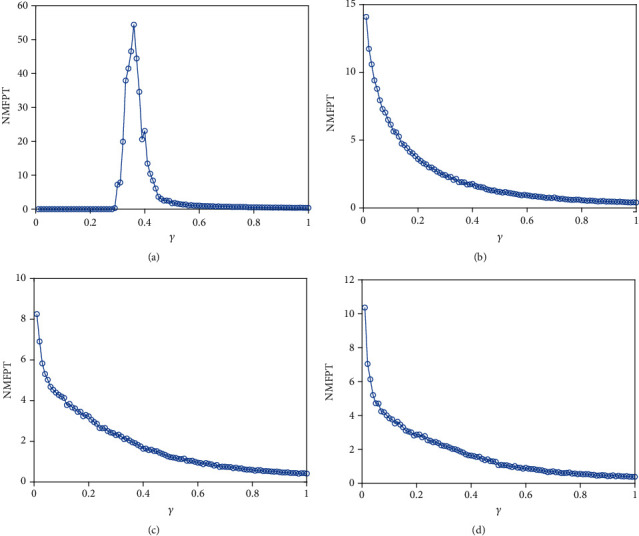
The network MFPT (NMFPT) about *γ*: (a) cc = 0; (b) cc = 0.6923; (c)cc = 0.7105; (d) cc = 0.7286.

**Figure 15:**
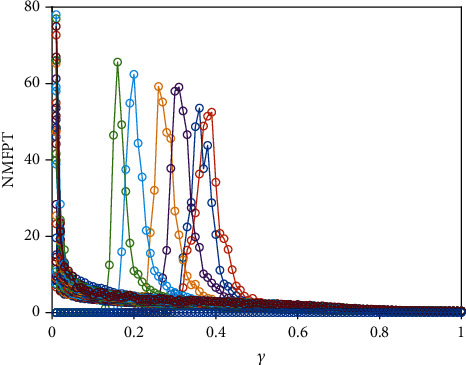
The network MFPT (NMFPT) about the clustering coefficient (cc) and noise strength.

Finally, we consider the network-organized FN model (18) with noise, and we mainly think about the effect of noise strength and the clustering coefficient on the switch of the steady state in the following. Although noise can induce the toggle switch [13, 27], how the noise affects the switch on the network remains to be solved. Now, we consider the switch of the steady state under different conditions. The switch does not occur when the noise strength is small (Figure 10(a)). The switch between the firing state and the resting state begins when the noise strength is larger (Figure 10(b)). Meanwhile, the switching frequency becomes faster and faster with the increase of noise strength (Figures 10(c) and 10(d)), which is the same with the FN model without network. Also, the clustering coefficient plays a vital role in the type of the switch (Figures 11–13). From Figure 11, the four regions always keep in a same state when *y* = 0.1 (Figure 11(a)), which represents four short-term memories that exist simultaneously. And the short-term memories may be disturbed when external stimulus increases (Figures 11(b)–11(d)). Namely, the higher clustering coefficient could induce the greater impact of noise on the neuron and make memories hazy (Figure 12); even a short-term memory never occurs (or memory disorder) (Figure 13). From Figure 13, the neurons remain in the firing state (Figure 13(a)) or resting state (Figure 13(b)) when the external stimulus is weak. Although a short-term memory occurs when the external stimulus is stronger, the short-term memory is weaker (Figure 13(c)) or hazy (Figure 13(d)). Also, we obtain the NMFPT about noise strength and the clustering coefficient (Figures 14 and 15). From Figure 14, the NMFPT increases firstly and decreases later when cc is small (Figure 14(a)), and the NMFPT decreases with noise strength when cc is larger than a critical value (Figures 14(b)–14(d)). Namely, noise strength and the clustering coefficient are employed together in the range of NMFPT (Figure 14). Meanwhile, the NMFPT is different due to cc (Figure 15), which means the clustering coefficient and the noise strength should have their own rangeability.

## 5. Conclusion

Although short-term memory attributes to a fixed point attractor, the role of a fixed point attractor was seldom investigated. In this paper, we investigate the role of both external stimulus and the clustering coefficient in short-term memory and show the role of a fixed point attractor. Firstly, we find neurons keep their steady state (resting or firing state) without external stimulus. Namely, no short-term memory activities occur when the system is always in a fixed point attractor (the resting state or firing state). Then, we study the effect of noise on the switch of the steady state in the bistable FN model and show that the MFPT is an important measurement tool to determine whether a short-term memory is lost or another one short-term memory occurs. Meanwhile, we find that the more intense the stimulus, the easier the short-term memory switch, and MFPT is the retention of short-term memory. These above results mean a fixed point attractor is a specific storage area for the short-term memory.

In general, a short-term memory should be kept for a while, rather than quickly disappear, which means that an appropriate stimulus is necessary to maintain a short-term memory. Furthermore, we find that the existence of a short-term memory or two short-term memories is possible in the brain. And we illustrate that the modest clustering coefficient and noise are necessary to maintain memories, and obtain NMFPT about the clustering coefficient and the noise strength. Finally, we find that the switching frequency becomes faster and faster with the increase of noise strength, and the higher clustering coefficient could induce the greater impact of noise on the neurons and make memories hazy. Namely, the modest clustering coefficient and noise strength are necessary for the maintaining of short-term memories. Also, it is found that some neurons are necessary to maintain short-term memory, rather than a neuron.

## Data Availability

All the data used to support the findings of this study are found within the article.
